# Urinary amino acids patterns enlighten new predictive models for extensive renal proximal tubular injuries and kidney function

**DOI:** 10.1515/jtim-2026-0049

**Published:** 2026-10-23

**Authors:** Xiaoxiao Shi, Yuan Dong, Wei Luo, Zuoxian Hou, Peng Xia, Wenling Ye, Ling Qiu, Limeng Chen

**Affiliations:** Department of Nephrology, Peking Union Medical College Hospital, Chinese Academy of Medical Science and Peking Union Medical College, Beijing, China; State Key Laboratory of Complex Severe and Rare Diseases, Beijing, China; Department of Clinical Laboratory, Peking Union Medical College Hospital, Chinese Academy of Medical Science and Peking Union Medical College, Beijing, China

**Keywords:** urinary amino acids, amino acid transporter, proximal tubular dysfunction, machine-learning diagnostic model

## Abstract

**Background and Objectives:**

The kidney plays a crucial role in amino acids (AAs) retention and excretion through AA transporters located in the proximal tubules. The impairment of these transporters results in aminoaciduria, one of the features of extensive proximal tubular (PT) dysfunction, namely renal Fanconi syndrome (FS). This study aimed to explore the clinical application of urinary AAs excretion profile and investigate their associations with renal function and other PT dysfunction.

**Methods:**

We enrolled 157 patients with PT dysfunction and detected urinary AAs levels by liquid chromatography-tandem mass spectrometry (LC-MS/MS). Machine learning models were used to assess diagnostic values of urinary AAs in renal FS and renal function decline, with randomized search to optimize parameters and evaluate algorithm performance.

**Results:**

The patients enrolled had a median age of 47 (35-60) years and a median eGFR of 92.8 (50.0-115.7) mL/min/1.73 m^2^, with primary Sjӧgren’s syndrome as the most common identified primary cause. We mapped etiology-specific urinary AAs excretion profiles. The most susceptible urinary AAs were citrulline (Cit) and threonine (Thr) transported by SLC6A19 (B^0^AT1), while arginine (Arg), aspartate (Asp) and histidine (His) were the least affected. Diagnostic models of renal FS achieved area under curve (AUC) values of 0.81 (XGBoost) and 0.82 (RF) based on all 21 urinary AAs, outperforming traditional clinical indicators. An optimization model based on four urinary AAs (Cit, phenylalanine [Phe], asparagine [Asn] and Asp) showed similar diagnostic performance (AUC 0.88). The urinary proline (Pro) level was independently correlated with eGFR < 60 mL/min/1.73 m^2^ (OR = 2.44, 95% CI: 1.53-3.91, *P* < 0.001). We found significant associations between abnormal urinary AAs excretion and hypouricemia or hypokalemia, particularly in the male patients.

**Conclusions:**

We mapped the etiology-specific urinary AAs excretion atlases in patients with PT injuries, and established a fine and simplified machine-learning model based on only four urinary AAs to diagnose extensive PT dysfunction noninvasively and conveniently. Significant associations of aminoaciduria with the other PT dysfunction showed gender differences.

## Introduction

Amino acids (AAs), the building blocks of life, serve various essential functions in protein synthesis, neurotransmission, acid-base balance, and intracellular energy metabolism.^[1]^ Dysregulations of AAs levels *in vivo* contribute to the pathogenesis of cancer, obesity, and diabetes.^[2]^ The kidney plays a crucial role in the excretion and reabsorption of AAs majorly facilitated by SLC family amino acid (AA) transporters, primarily located in the S1 segment of proximal tubules.^[1,3]^ Each AA transporter is responsible for transporting specific AAs between urine and proximal tubular (PT) cells, and there are four most important renal AA transporter complex proteins including the luminal Na^+^-dependent neutral AA transporters of the SLC6 family such as SLC6A19 (Broad neutral AA transporter 1, B^0^AT1) and SLC6A20 (Sodium/iminoacid transporter 1, SIT1) for reabsorbing neutral AAs,^[4,5]^ SLC7A9 (b^0,+^-type AA transporter 1, b^0,+^AT1) with accessory protein SLC3A1 (neutral and basic AA transport protein, rBAT) responsible for transporting cysteine (Cys) and the dibasic AAs (ornithine, Orn; lysine, Lys; and arginine, Arg) (alternate name the COLA transporter), the symporter SLC1A1 (Excitatory AA transporter 3, EAAT3) transporting anionic AAs such as aspartic acid (Asp) and glutamic acid (Glu), and H^+^ dependent SLC36A1/2 (Proton-assisted AA transporter 1/2, PAT1/2) transporting glycine (Gly), alanine (Ala) and proline (Pro) (Figure 1).^[6, 7, 8, 9]^ The dysfunction of these AA transporters leads to reduction of renal resorption of AAs and elevation of urinary AAs excretion, namely aminoaciduria.^[10]^ Aminoaciduria can be part of extensive renal PT transport dysfunction, known as a disease called renal Fanconi syndrome (FS), clinically manifested as glycosuria, hyperphosphaturia with or without hypophosphatemia, hyperuricosuria with or without hypouricemia, hypokalemia, and type II proximal renal tubular acidosis (RTA) beyond aminoaciduria. ^[11,12]^ Similar to renal FS, aminoaciduria can be caused by hereditary diseases, autoimmune diseases, drugs, plasmacyte diseases, and other rare reasons.^[11]^

**Figure 1:**
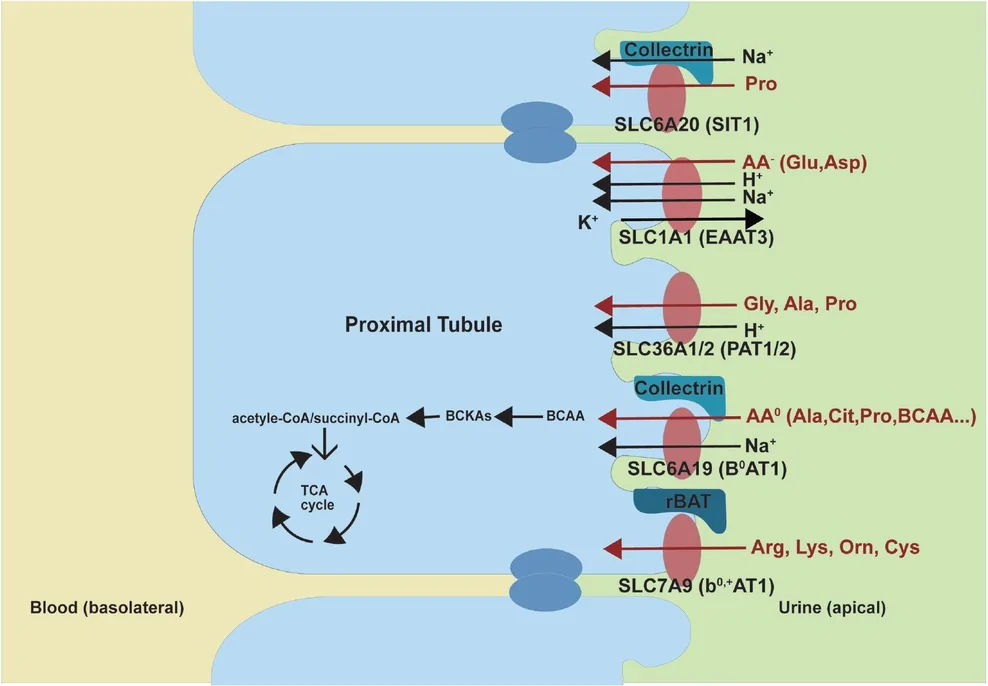
Simplified scheme of identified amino acid transporters on the apical side of renal proximal tubules and possible amino acids metabolism pathway in proximal tubular cells. Collectrin is the accessory protein for SLC6A19 (B^0^AT1) as a transporter complex, and SLC3A1 (rBAT) is the same for SLC7A9 (b^0,+^AT1). AA0: neutral amino acids; AA-: acidic (anionic) amino acids; Gly: glycine; Pro: proline; Ala: alanine; Glu: glutamate; Asp: asparate; Cit: Citrulline; Arg: arginine; Lys: lysine; Orn: ornithine; Cys: cystine; BCAAs: branched chained amino acids, including leucine, valine, isoleucine; BCKAs: branched-chain α-keto acids; TCA: Tricarboxylic Acid Cycle. The Schematic diagram illustrated the concept described by Makrides *et al*.^[3]^

Urinary AAs can be detected accurately by liquid chromatography-tandem mass spectrometry (LC-MS/MS) in recent years. However, the precise clinical interpretation of urinary AAs levels, including the characteristics and severity of abnormal urinary AAs excretion among different diseases, still remain unclear and worth further study. Certain AAs like the branched-chain AAs (BCAAs) including valine (Val), leucine (Leu) and isoleucine (Ile) may serve as an energy source in PT cells.^[13]^ BCAAs absorbed by PT cells can be metabolized to branched-chain α-keto acids (BCKAs) and further to acetyl-coenzyme A (acetyl-CoA) /succinyl-CoA which enter tricarboxylic acid cycle and provide energy (Figure 1).^[14]^ Previous studies have suggested the catabolism of BCAAs in PT cells could alleviate PT injuries and preserve renal function in both acute kidney injury (AKI) and chronic kidney disease (CKD) mouse models. Additionally, there is evidence suggesting that BCAA metabolism is downregulated in patients with CKD.^[13,15,16]^ Thus, aminoaciduria may not only be an outcome but also a participating and exacerbating factor in PT injuries. The clinical application values of urinary AAs detection beyond being a marker for dysfunction of AA transporters in PT cells worth exploring.

Intriguingly, previous studies have shown aminoaciduria and AAs transporting in proximal tubules may have relations with the other PT transporters and substrates transporting. Mutations in some AA transporter genes may result in not only aminoaciduria but also some other PT dysfunction like glucosuria and hyperphosphaturia,^[17,18]^ and vice versa, the other PT dysfunction such as glycosuria caused by mutations of sodium-glucose cotransporter 2 (SGLT2) can also lead to aminoaciduria.^[19]^ These indicate potential connections between the transporting of AAs and the other substrates in proximal tubules, which requires more studies especially in patients with PT injuries of nongenetic etiologies. And urinary AAs detection may become a noninvasive and convenient method in diagnosing extensive PT dysfunction (namely renal FS) instead of traditional cumbersome steps by repeatedly drawing blood and collecting urine.

In this study, we described the urinary AAs excretion profile of patients with different etiologies of PT dysfunction, tried to establish a simplified model for diagnosing renal FS based on urinary AAs levels, and investigated the potential associations between abnormal urinary AAs excretion with renal function and the other PT transport defects.

## Materials and methods

### Patients and groups

All adult patients (older than 18 years old) with urinary AAs detection results and PT dysfunction in Peking Union Medical College Hospital (PUMCH) from November 2021 to June 2023 were enrolled. The diagnosis of PT dysfunction required the presence of one of these five criteria corresponding to different PT transporters dysfunction:^[12,20]^ (I) Normoglycemic glycosuria; (II) Aminoaciduria; (III) Hypophosphatemia and hyperphosphaturia; (IV) Hypouricemia, which should exclude other factors leading to it such as urate-lowering drugs, malnutrition, severe hepatic injury, syndrome of inappropriate secretion of antidiuretic hormone, diabetes mellitus, pregnancy, and intractable diarrhea; and (V) Proximal RTA. Renal FS was defined as follows:^[12,20]^ (1) the existence of at least three of the five items or (2) the existence of at least two of the five items and evidence of PT damages in kidney pathology. Exclusion criteria were as follows: (1) patients without renal PT dysfunction; (2) patients with conditions of abnormal amino acids absorption, generation or consumption, including intravenous amino acids infusion, severe malnutrition, malabsorption, severe liver dysfunction or liver cirrhosis except hepatolenticular degeneration, severe infections, malignant tumors, and pregnancy;^[21]^ (3) patients without consent.

### Clinical data collection

The medical records of the enrolled patients were reviewed for demographic features, diagnostic information, and laboratory and kidney pathologic findings. Laboratory tests included routine tests of blood and urine, serum creatine, serum and urinary electrolytes (potassium, phosphate, calcium, sodium, magnesium), uric acid levels, and arterial blood gas analysis.

### Evaluation of renal tubular functions

#### Detection of urinary AAs

Morning midstream urine samples from enrolled participants who underwent fasting overnight were collected.^[22]^ Urine samples, calibrators, and quality control samples were precipitated by adding working solution and centrifuged at 16,200 ×*g* for 10 min. The clear supernatant was used for urinary AAs detection by LC-MS/MS and adjusted by its urinary creatinine level.

#### Hyperphosphaturia

Hyperphosphaturia was defined by one of the following standards. (1) increased urinary fractional excretion of phosphate (FEPO4 = [urine phosphate × plasma creatinine] / [plasma phosphate × urine creatinine] × 100%, > 20%);^[23]^ (2) increased 24-hour phosphorus excretion (> 100 mg/d) in the condition of hypophosphatemia; (3) decreased renal tubular reabsorption of phosphate (TmP/GFR < 0.77 mmol/L).^[24]^

#### Identification of RTA types

Patients were evaluated by arterial blood gas analysis, urinalysis, and urinary electrolyte measurements. Hyperchloremic metabolic acidosis was diagnosed when serum pH was < 7.35 and serum bicarbonate levels were < 24 mmol/L with a normal anion gap. If such a patient simultaneously had urine pH > 5.5, positive urine anion gap, and hypokalemia, distal RTA was considered. The RTA type was further validated by the sodium bicarbonate infusion test with sodium bicarbonate infusion till the serum bicarbonate reached 24 mmol/ L and fractional excretion of bicarbonate (FEHCO3) measured by the formula [urine bicarbonate × plasma creatinine] / [plasma bicarbonate × urine creatinine] × 100%. The proximal RTA was confirmed when FEHCO3 was more than 10%-15%, and if less than 5%, distal RTA was diagnosed.

### Statistical analysis

Continuous variables were reported as mean ± standard deviation or median (interquartile range [IQR]) as appropriate. Categorical variables were expressed as percentages. Comparisons of the clinical characteristics between patients with different etiologies were performed by one-way analysis of variance (ANOVA), Kruskal-Wallis ANOVA, or *Chi-square* test as appropriate. Comparisons of the ratios of abnormal urinary excretion of each AA between the patients with and without a specific PT dysfunction (including hypouricemia, hypokalemia, hypophosphatemia, and normoglycemic glycosuria) were performed by *chi-square* test. The associations between urinary AAs excretion levels and serum biochemical parameters (uric acid, potassium, phosphate) were evaluated using Pearson’s correlation or Spearman’s correlation coefficient assessments. Multiple logistic regression analysis was performed to investigate the influencing factors of estimated glomerular filtration rate (eGFR) < 60 mL/min/1.73 m^2^. In the logistic regression analysis, the variable age with a non-normal distribution was z-score normalized, and the variable etiology was transformed into a dummy variable with the etiology “plasmacyte disease” as reference for the patients with plasmacyte disease in our cohort showed the most severe proximal tubular dysfunction.

A *P*-value of < 0.05 was considered as statistically significant. Analyses were performed using the Statistical Package for the Social Sciences (SPSS, version 20.0, SPSS Inc., Chicago, IL, USA) and the GraphPad Prism 6.0 (GraphPad Software Inc., CA, USA). The hierarchical clustering analyses were performed by Ward’s minimum variance method by R language (version 4.3.1) in an unsupervised learning manner.

To evaluate the diagnostic values of urinary AAs and traditional clinical indicators (serum potassium, serum phosphate, serum uric acid, and urinary glucose) in renal FS, we established a machine learning algorithm for binary classification using Random Forest Regressor (RFR) and eXtreme Gradient Boosting (XGBoost) in Python (version 3.8.8) with a training set and a testing set (8:2). Machine learning methods were employed to continuously simplify the parameters of urinary AAs, selecting the most efficient and effective diagnostic model based on the changes in area under curve (AUC), recall, accuracy, precision, F1 score, and mean squared error (MSE) during the optimization process. We used Randomized search to find out the best model hyperparameters, calculated feature importance for contribution evaluation, and Receiver Operating Characteristic (ROC) curves to evaluate the performance of classification algorithms. To mitigate overfitting, Random Forest (RF) and XGBoost models were optimized *via* Bootstrap sampling for diverse tree construction, 10-fold cross-validated hyperparameter tuning, stepwise feature selection to reduce complexity, and the inherent regularization mechanisms of these ensembles methods. Predictive models of eGFR < 60 mL/min/1.73 m^2^ with urinary AAs levels were also established through machine learning with the same method as above.

## Results

### Clinical characteristics

A total of 157 patients with PT dysfunction who had detected urinary AA levels were identified and the most common causes were autoimmune diseases (19.7%), hereditary diseases (11.5%), drug-induced kidney injuries (10.8%), plasmacyte diseases (5.1%), and tumor-induced osteomalacia (TIO) (20.4%). Among them, primary Sjögren’s syndrome (pSS) was the most common autoimmune disease, and the most frequent drugs-induced etiologies involved traditional Chinese medicine, antiviral drugs, and chemotherapeutic drugs (Table 1). The median age of all the patients was 47 (35-60) years, with 59.8% females. The median serum creatinine and eGFR levels were 77 (58-113) μmol/L and 92.8 (50.0-115.7) mL/min/1.73 m ^2^, respectively. The most common PT dysfunction included aminoaciduria (86.6%), hypophosphatemia (51.3%), and hypokalemia (43.1%) (Table 2).

**Table 1:** Etiology of the 157 patients with proximal tubular dysfunction in this study

Etiology	*n* (%)
Autoimmune disease	31 (19.7)
pSS	26 (16.6)
Undifferentiated connective tissue diseases	3 (1.9)
Systemic scleroderma	2 (1.3)
Hereditary disease	18 (11.5)
Hepatolenticular degeneration	7 (4.5)
Familial renal diabetes mellitus	4 (2.5)
Batter syndrome	2 (1.3)
Gitelman syndrome	2 (1.3)
Other hereditary diseases*	3 (1.9)
Drug-induced	17 (10.8)
Traditional Chinese medicine	7 (4.5)
Adefovir	3 (1.9)
Tenofovir	2 (1.3)
Other drugs^#^	5 (3.2)
Plasmacyte disease	8 (5.1)
MGRS	4 (2.5)
Multiple myeloma	3 (1.9)
Heavy chain deposition disease	1 (0.6)
Other related causes	45 (28.7)
Tumor induced osteomalacia	32 (20.4)
Primary hyperparathyroidism	3 (1.9)
Primary aldosteronism	3 (1.9)
Tubulointerstitial nephritis and uveitis	2 (1.3)
Membranous nephropathy	2 (1.3)
IgA nephropathy	1 (0.6)
Diabetic kidney disease	1 (0.6)
Chronic myeloid leukemia	1 (0.6)
Unknown causes	38 (24.2)

*Other hereditary diseases included autosomal dominant hypophosphatemic rickets (*n* = 1), SLC4A1 gene mutation (*n* = 1), and McCune-Albright syndrome (*n* = 1). ^#^Other drugs included 5-fluorouracil (*n* = 2), furmonertinib (*n* = 1), pembrolizumab (*n* = 1) and gemcitabine (*n* = 1). pSS: primary Sjögren’s syndrome; MGRS: monoclonal gammopathy of renal significance.

**Table 2:** Comparisons of clinical characteristics among the patients with different etiologies

Items	All cases (*n* = 157)	pSS (*n* = 26)	Drug-induced (*n* = 17)	Plasmacyte disease (*n* = 8)	TIO (*n* = 32)	*P*
Demographic characteristics						
Female	94 (59.8)	26 (100)	6 (35.3)	4 (50.0)	12 (37.5)	< 0.001
Age	47 (35-60)	42 (35-58)	60 (39-63)	61 (52-69)	47 (35-57)	0.025
Renal function						
Serum creatinine, median (μmol/L)	77 (137, 58-113)	98 (26, 62-123)	108 (15, 76-181)	136 (8, 112-167)	65 (25, 52-76)	0.065
eGFR (mL/min/1.73 m^2^)	92.8 (137, 50.0-115.7)	69.1 (26, 42.6-104.5)	64.6 (15, 33.5-91.5)	46.8 (8, 27.9-53.5)	105.0 (25, 94.8-118.1)	< 0.001
eGFR<60 mL/min/1.73 m^2^	41/137 (29.9)	12 (46.2)	7/15 (46.7)	7 (87.5)	3/25 (12.0)	< 0.001
Tubular dysfunction						
Aminoaciduria	136 (86.6)	25 (96.2)	16 (94.1)	7 (87.5)	29 (90.6)	0.945
Hypophosphatemia	77/150 (51.3)	9 (34.6)	5/15 (33.3)	5 (62.5)	31/31 (100)	< 0.001
Serum phosphorus (mmol/L)	0.93 ± 0.32 (150)	0.97 ± 0.25 (26)	1.00 ± 0.31 (15)	1.00 ± 0.48 (8)	0.66 ± 0.20 (31)	< 0.001
Hypokalemia	66/153 (43.1)	19 (73.1)	4 (23.5)	5 (62.5)	7/30 (23.3)	< 0.001
Serum potassium (mmol/L)	3.7 ± 0.5 (153)	3.6 ± 0.7 (26)	3.9 ± 0.5 (17)	3.9 ± 0.7 (8)	3.9 ± 0.4 (30)	0.166
Normoglycemic glycosuria	52/152 (34.2)	6 (23.1)	3/15 (20.0)	5 (62.5)	15 (46.9)	0.044
Hypouricemia	42/136 (30.8)	15 (57.7)	6/14 (42.9)	6 (75.0)	3/24 (12.5)	< 0.001
Serum uric acid (μmol/L)	262 (136, 161-336)	161 (26, 120-275)	194 (14, 151-280)	140 (8, 105-187)	336 (24, 258-445)	0.004
Hyperphosphaturia	18/68 (26.5)	1/8 (12.5)	1/7 (14.3)	0/4 (0)	2/15 (13.3)	0.906
Proteinuria	32/135 (23.7)	4 (15.4)	4/15 (26.7)	3 (38.0)	14 (44.0)	0.084
Renal tubular acidosis	23/121 (19.0)	1/22 (4.5)	5/15 (33.3)	3/7 (42.9)	3/28 (10.7)	0.158
Plasma pH	7.38 (85, 7.32-7.41)	7.38 (12, 7.31-7.42)	7.37 (7, 7.34-7.38)	7.37 (5, 7.31-7.38)	7.36 (28, 7.30-7.41)	0.773
Plasma HCO3^-^ (mmol/L)	20.2 ± 4.7 (74)	18.7±3.4 (12)	20.2 ± 5.2 (5)	18.3 ± 5.6 (4)	19.7 ± 5.1 (28)	0.522

pSS: primary Sjögren’s syndrome; TIO: tumor-induced hypophosphatemia osteomalacia; IQR: interquartile range; SD: standard deviation; eGFR: estimated glomerular filtration rate.

The patients with different etiologies presented with etiology-specific patterns of clinical manifestations especially in tubular dysfunction (*P* < 0.05). The pSS patients had a higher ratio of females. The patients with plasmacyte diseases were characterized by older age, worse renal functions, and a higher prevalence of normoglycemic glycosuria. The patients with TIO had a lower ratio of hypouricemia. Both pSS and plasmacyte diseases had higher ratios of hypokalemia, while the patients with TIO or plasmacyte diseases had higher prevalences of hypophosphatemia (Table 2).

### Urinary AAs excretion atlases and clustering analyses

The urinary BCAAs levels were significantly elevated in the patients with eGFR decline ( < 60 mL/min/1.73 m^2^) compared to the patients with eGFR≥60 mL/min/1.73 m^2^ (Figure 2A). The most susceptible urinary AAs were citrulline (Cit, 56.7%) and threonine (Thr, 52.2%), with the most common affected AA transporter as SLC6A19. The least susceptible AAs were Arg (13.4%), Asp (16.6%), and histidine (His, 24.8%) (Supplementary Table S1). The atlas of urinary AAs profile indicated that the patients with more extensive abnormal urinary AAs excretion usually had higher urinary AA excretion ratios (Figure 2B, G). According to the degrees and extents of abnormal urinary AAs excretion, the hierarchical clustering analyses showed potential associations between specific AAs such as Cit and Pro transported by SLC6A19 and SLC36A1/2, respectively (Figure 2B).

**Figure 2:**
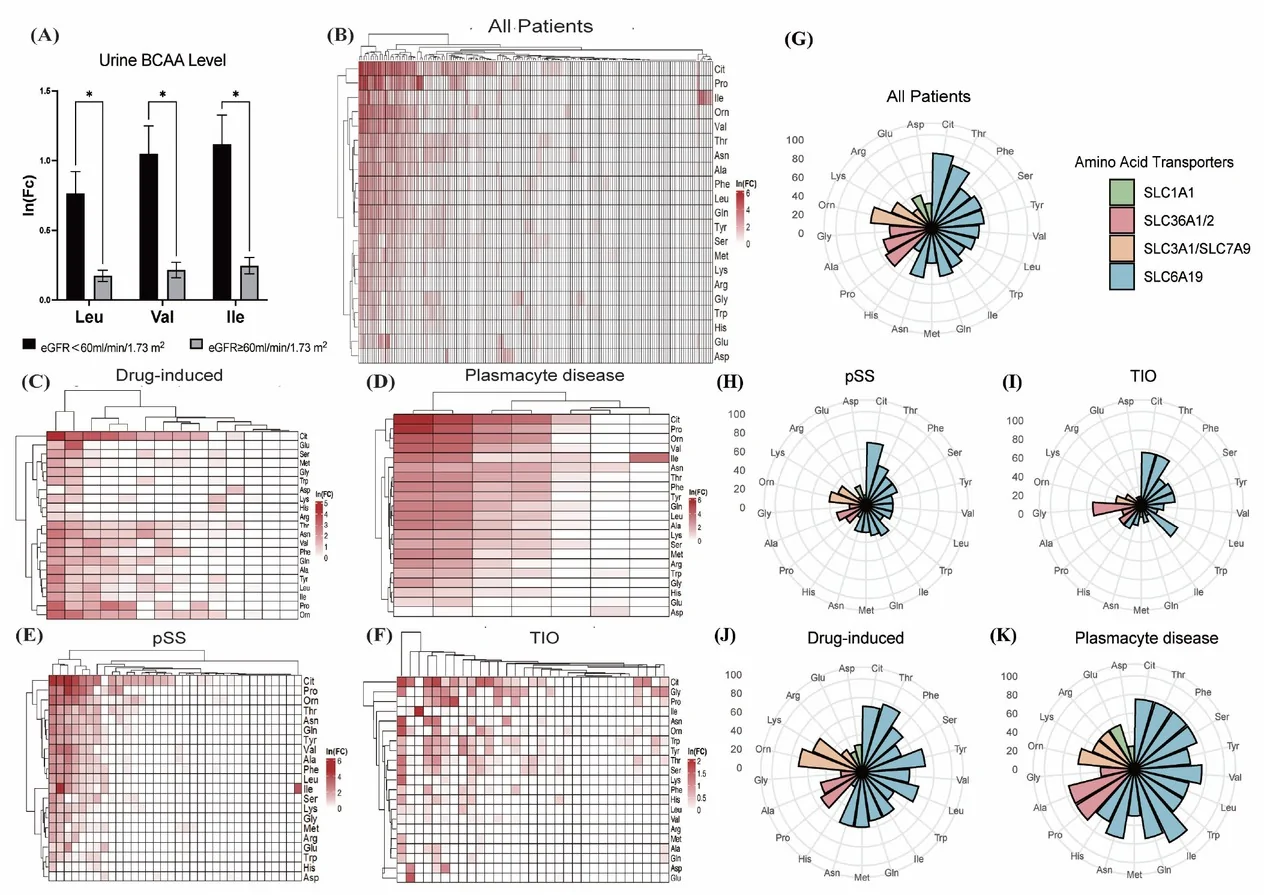
The atlases and clustering analyses of abnormal urinary amino acids excretion in the patients with proximal tubular dysfunction. (A) Urinary BCAAs levels increased significantly in the patients with eGFR decline (< 60 mL/min/1.73 m2) compared with the patients with eGFR ≥ 60 mL/min/1.73 m^2^; ^*^P < 0.001. (B-F) The clustering analyses of urinary amino acids excretion in all the patients (B) and the patients with different etiologies (C-F). The fold change was calculated by dividing each urinary amino acid level by its upper limit of the normal range. The hierarchical clustering analyses were carried out according to the patterns and degrees of abnormal urinary amino acid excretion using Ward’s minimum variance method by R language in unsupervised learning. (G-K) The atlases of abnormal urinary amino acids excretion in all the patients (G) and the patients with different etiologies (H-K). The lengths of the bands in the atlases were determined by the prevalences of the abnormal urinary excretion of each amino acid. The colors of the bands represented different amino acid transporters which contributed to the transporting of different amino acids across the luminal membranes of renal proximal tubular cells. FC: fold change; BCAAs: branched-chain amino acids; Ala: alanine; Arg: arginine; Asn: asparagine; Asp: aspartic acid; Cit: citrulline; Gln: glutamine; Glu: glutamic acid; Gly: glycine; His: histidine; Ile: isoleucine; Leu: leucine; Lys: lysine; Met: methionine; Orn: ornithine; Phe: phenylalanine; Pro: proline; Ser: serine; Thr: threonine; Trp: tryptophan; Tyr: tyrosine; Val: valine; pSS: primary Sjögren’s syndrome; TIO: tumor-induced hypophosphatemia osteomalacia.

The patients with different etiologies showed etiology-specific patterns of urinary AAs excretion. Both Cit and Thr were significantly increased in the urine in all the groups with different etiologies. Urinary excretion of Orn, Ile, glutamine (Gln), Pro, methionine (Met), and Lys were more commonly increased in the patients with plasmacyte diseases or drug-induced PT dysfunction. Abnormal urinary excretion of Ala and Arg were more commonly detected in the patients with plasmacyte diseases (Figure 2C-F, H-K; Supplementary Table S1).

### Establishing simplified diagnostic models of renal FS with urinary AAs through machine learning

Diagnostic models for renal FS were established using XGBoost or RF machine learning algorithms on a training and testing set (8:2), incorporating various combinations of traditional clinical indicators (serum potassium, serum phosphate, serum uric acid, and urinary glucose) and urinary AAs (Figure 3 I-L). The models using all the 21 urinary AAs showed AUC values of 0.81 in XGBoost algorithm and 0.82 in RF algorithm respectively, which were both better than the models using traditional clinical indicators (AUC 0.74 and 0.78, respectively), and achieved similar diagnostic values as the models combining both the traditional clinical indicators and urinary AAs (AUC 0.84 and 0.82, respectively) (Figure 3I-L). Furthermore, Randomized search for hyperparameter optimization was carried out in various combinations of urinary AAs using XGBoost or RF machine learning algorithms (Figure 3A-H). By comparing the final simplified models based on multiple parameters (AUC, F1, Accuracy, MSE, Precision, Recall), an optimal simplified model with four urinary AAs (Cit, phenylalanine[Phe], asparagine[Asn] and Asp) though XGBoost algorithm showed an equivalent diagnostic value in renal FS with an AUC value of 0.88 (Figure 3 A-J).

**Figure 3:**
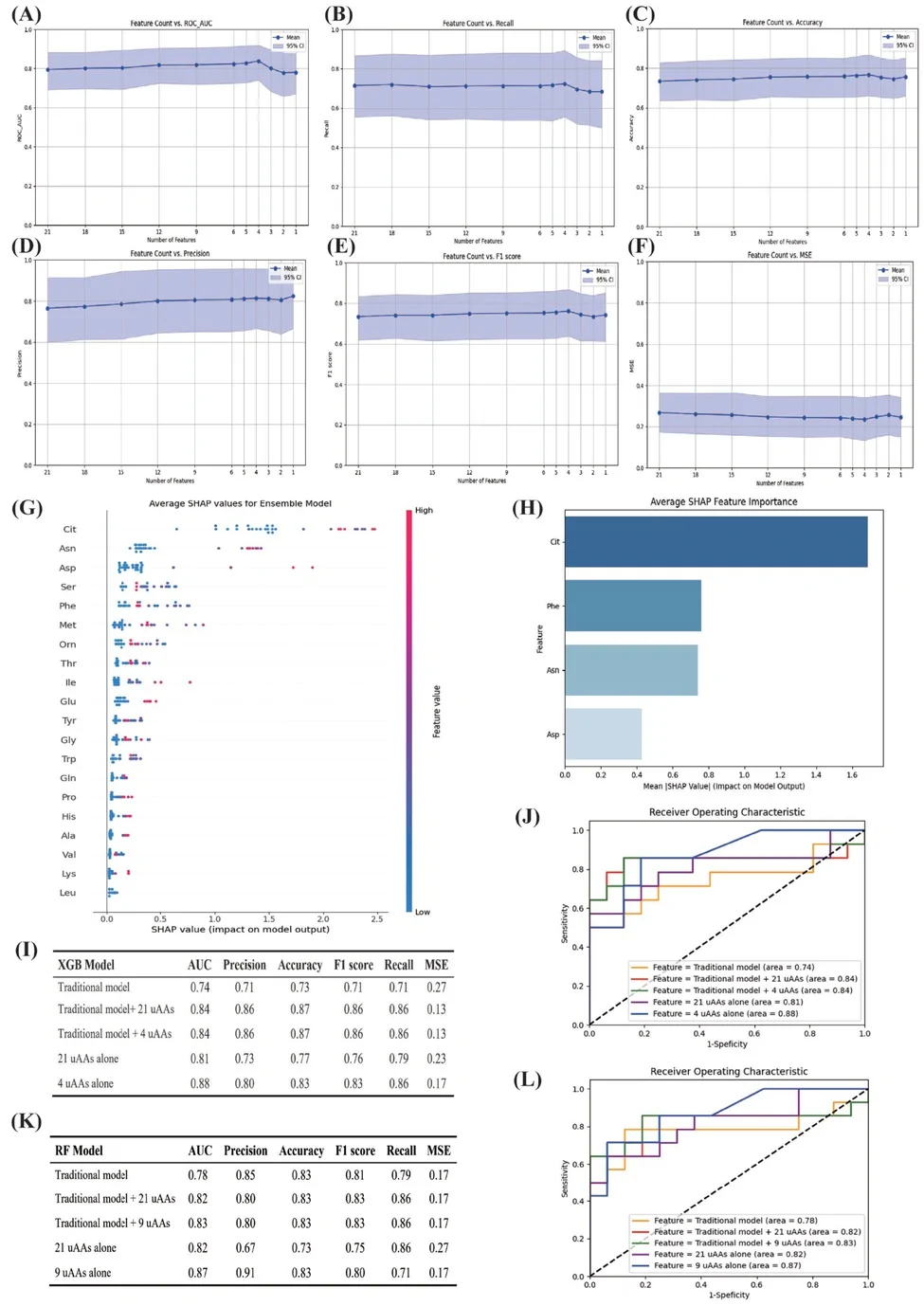
The diagnostic models of renal FS with uAAs levels through machine learning. The models were constructed using the XGBoost or RF machine learning algorithm with different combinations of traditional clinical indicators (serum potassium, serum phosphate, serum uric acid, and urine glucose) and uAAs. (A-F) The changes in the model performance metrics including AUC (A), Recall (B), Accuracy (C), Precision (D), F1 Score (E), and MSE (F) during optimizing model features with different combinations of uAAs using XGBoost machine learning algorithm. The optimizing process showed that only four uAAs (Cit, Phe, Asn and Asp) could reach a similar diagnostic value in renal FS as using all the 21 uAAs. (G) The SHAP summary plot of the XGBoost model with the 21 uAAs. Each dot represents an individual patient from the dataset. Red represents the higher SHAP value of specific features; blue represents the lower SHAP value of specific features. The higher the SHAP values, the greater the risk of renal FS. (H) The SHAP summary plot of the XGBoost model with the 4 uAAs. (I-J) The model performance metrics of different models in diagnosing renal FS with XGBoost (I-J) or RF (K-L) machine learning algorithm. The traditional models were established with traditional clinical indicators including serum potassium, serum phosphate, serum uric acid, and urine glucose. The 21 uAAs models were established with all the 21 uAAs. that could be detected in this study. The 4 uAAs included Cit, Phe, Asn, and Asp, which were obtained by the above optimization process using XGBoost machine learning algorithm. The nine uAAs included Cit, Asn, Phe, Thr, Asp, Orn, Ala, Gln, Pro, which were obtained by the optimization process using RF machine learning algorithm. FS: Fanconi syndrome; uAAs: urinary amino acids; XGBoost: eXtreme Gradient Boosting; RF: random forest; AUC: area under the curve; MSE: mean squared error; SHAP: SHapley Additive exPlanation; Cit: citrulline; Phe: phenylalanine; Asn: asparagine; Asp: aspartic acid; Thr: threonine; Orn: ornithine; Ala: alanine; Gln: glutamine; Pro: proline.

### Associations between abnormal urinary AAs excretion and renal insufficiency

#### Logistic regression analysis of influence factors for renal insufficiency

Logistic regression analysis showed that urinary Pro level, etiology, and age were associated with eGFR < 60 mL/min/1.73 m^2^ (*P* < 0.05). Multiple logistic regression analysis showed that the urinary Pro level independently correlated with eGFR < 60 mL/min/1.73 m^2^ (OR = 2.44, 95% CI:1.53-3.91, *P* < 0.001) when adjusted by age and etiology (Table 3).

**Table 3:** Univariate and multivariate analyses of independent factors associated with eGFR < 60 mL/min/1.73 m^2^

Indicators	Univariant			Multivariate		
	OR	95% CI	*P*-value^#^	OR	95% CI	*P*-value^#^
Urinary proline level	1.92	1.44-2.58	< 0.001	2.44	1.53-3.91	< 0.001
Age (Z-score)	3.36	2.03-5.57	< 0.001	4.94	2.44-9.96	< 0.001
Etiology*						
Plasmacyte disease	Reference			Reference		
Primary Sjögren syndrome	0.122	0.013-1.142	0.065	0.384	0.028-5.189	0.471
Other autoimmune diseases	0.048	0.002-1.040	0.053	0.027	0.001-0.888	0.043
Drug-induced tubular dysfunction	0.125	0.012-1.282	0.080	0.134	0.009-2.024	0.147
Hereditary disease	0.010	0.001-0.189	0.002	0.070	0.002-1.964	0.118
TIO	0.095	0.009-0.985	0.049	0.113	0.007-1.745	0.118
Other related cause	0.006	0-0.108	0.001	0.015	0.001-0.348	0.009
Unknown causes	0.037	0.004-0.364	0.005	0.017	0.001-0.287	0.005

*Etiology was clustered into eight subclasses including primary Sjögren syndrome, other autoimmune diseases, hereditary disease, drug-induced tubular dysfunction, plasmacyte diseases, TIO, other related causes except for TIO, and unknown causes. In the multivariable logistic regression analysis, etiology was transformed as a dummy variable with plasmacyte disease as the reference group. ^#^ *P*-value was calculated using maximum likelihood estimation (MLE) in binary logistic regression. OR: odds ratio; CI: confidence interval; TIO: tumor-induced hypophosphatemia osteomalacia.

#### Predictive models of renal insufficiency with urinary AAs through machine learning

Predictive models for eGFR < 60mL/min/1.73 m^2^ were established with urinary AAs through XGBoost or RF machine learning algorithms with a training set and a testing set (8:2). The AUC values were 0.94 and 0.92 in XGBoost and RF algorithms, respectively (Supplementary Figure S1 I-J). Randomized search for hyperparameter optimization was also carried out in various combinations of urinary AAs, and an optimal simplified model with seven urinary AAs (Cit, Orn, Pro, Ile, tryptophan [Trp], Thr, and His) showed an equivalent predictive value with an AUC value of 0.94 through XGBoost machine learning algorithm (Supplementary Figure S1 A-J).

### Associations between abnormal urinary AAs excretion and the other PT dysfunction

To observe the associations between abnormal urinary AAs excretion with the other PT dysfunction, we compared the ratios of abnormal urinary AAs excretion between the patients with or without a specific PT dysfunction including hypouricemia, hypokalemia, hypophosphatemia, and normoglycemic glycosuria. For hypouricemia, the patients with hypouricemia (the Hypouricemia group) had significant higher ratios of abnormal urinary excretion of 14 AAs, including Cit, Asn, Tyr, Phe, Gln, Ile, Leu, Val, Orn, Lys, Arg, Ala, Pro, and Glu) than the patients without hypouricemia (the non-hypouricemia group) (Figure 4A, Table S2). It was interesting that the associations of urinary AA excretion levels with serum uric acid levels were more prominent in the male patients than the female patients. Almost all the urinary AAs levels except Gly were significantly associated with serum uric acid levels in the male patients, whereas only five urinary AAs (Ala, Asn, Gln, Cit, and Lys) showed similar significant associations (*P <* 0.05) in females (Figure 4B-D, Supplementary Table S3a and S3b). For hypokalemia, the ratios of abnormal urinary excretion of seven AAs, including Ala, Ile, Asn, Gln, Cit, Lys, Asp and Glu, were significantly higher in the patients with hypokalemia than the patients without hypokalemia (Supplementary Table S2). Urinary excretion of Ala, Pro, Gly, and Arg were significantly associated with serum potassium levels (Supplementary Table S4). The associations of urinary AAs excretion levels with serum potassium levels also showed a noteworthy gender difference which was more pronounced in males (Supplementary Table S3a and S3b). For hypophosphatemia, the ratios of abnormal urinary excretion of three AAs, including Gly, Thr, and Cit, were significantly higher in the patients with hypophosphatemia than the patients without hypophosphatemia (Supplementary Table S2). None of the urinary AAs excretion were significantly associated with serum phosphate levels (Supplementary Table S4). For normoglycemic glycosuria, the ratios of abnormal urinary AAs excretion did not differ between the patients with and without normoglycemic glycosuria (Supplementary Table S2).

**Figure 4:**
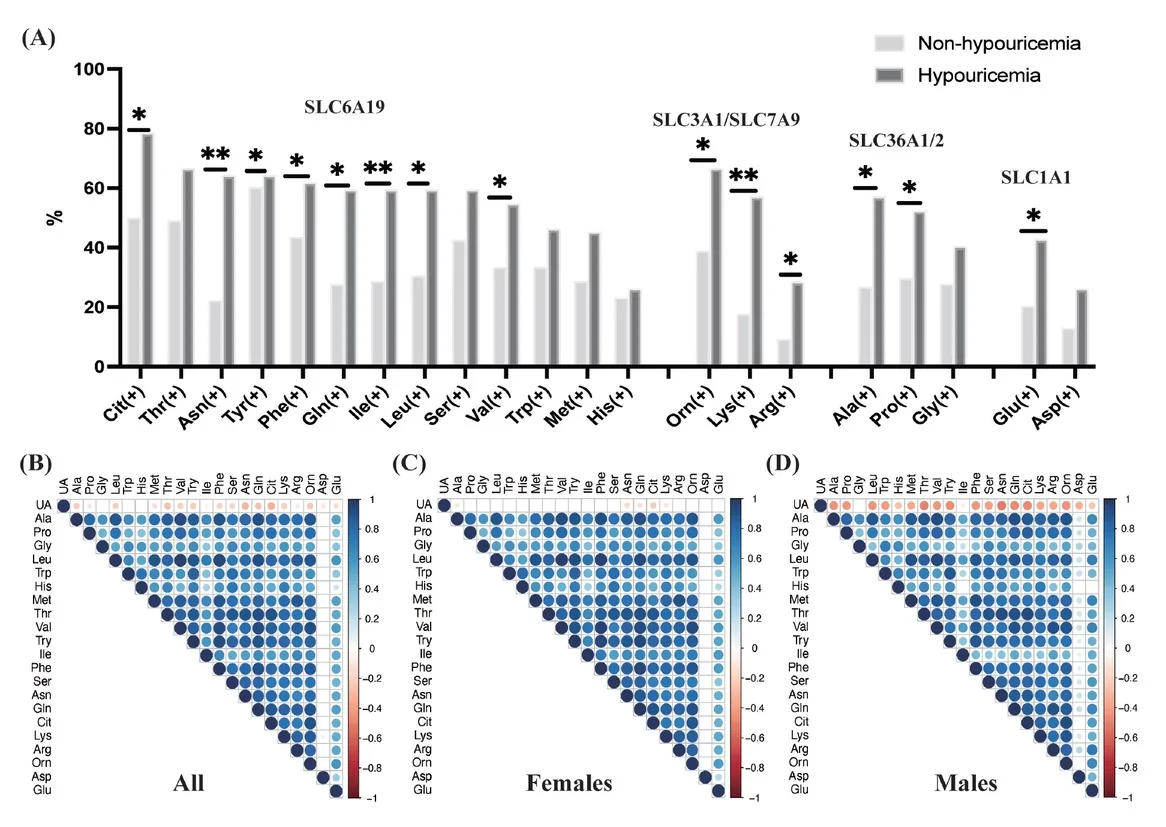
The associations of abnormal urinary amino acids excretion and hypouricemia in the patients with proximal tubular dysfunction. (A) Comparisons of the ratios of abnormal urine excretion of each amino acid between patients with and without hypouricemia (^*^P < 0.05; ^**^P < 0.001). (B-D) Correlation analyses of serum uric acid levels with urinary amino acid excretion levels in all patients (B), female patients (C) and male patients (D). The sizes and the colors of the dots represented the values of the correlation coefficient as shown in the sidebar, The correlation coefficients with no statistical significance (*P* > 0.05) were not shown in the figure. Ala: alanine; Arg: arginine; Asn: asparagine; Asp: aspartic acid; Cit: citrulline; Gln: glutamine; Glu: glutamic acid; Gly: glycine; His: histidine; Ile: isoleucine; Leu: leucine; Lys: lysine; Met: methionine; Orn: ornithine; Phe: phenylalanine; Pro: proline; Ser: serine; Thr: threonine; Trp: tryptophan; Tyr: tyrosine; Val: valine; UA: serum uric acid level.

## Discussion

In this study, we mapped the unique etiology-specific urinary AAs excretion atlases from 157 patients with PT dysfunction caused by various primary diseases and described the associations between aminoaciduria and decreased renal function. We also established a fine and simplified diagnostic model of renal FS with only four specific urinary AAs by machine learning, which was more accurate than models with traditional clinical indicators and showed a significant clinical application potential.

Only a few studies have explored urinary AAs excretion profiles in PT injuries caused by hereditary diseases.^[25, 26, 27]^ The clinical application values of urinary AAs in nonhereditary diseases remained unknown. To our limited knowledge, we mapped urinary AAs excretion profiles in the largest cohort of patients with PT injuries of various etiologies. We identified the most susceptible urinary AAs as Cit and Thr transported mainly by SLC6A19 among patients with different etiologies. It suggested the AA transporter SLC6A19 might be sensitive to various types of PT injuries and urinary Cit and Thr excretion levels might become potential indicators for renal PT injuries, a hypothesis that warrants further validation. We also observed etiology-specific patterns of urinary AAs excretion. For instance, Orn, Lys, and Arg transporting by the COLA transporter (SLC3A1/SLC7A9) were all more frequently affected in the patients with plasmacyte diseases, suggesting this transporter might be more susceptible to damages caused by light chain deposition in PT cells. However, it might just be a result of severe PT injuries as the patients with plasmacyte diseases in our cohort seemed to suffer more severe kidney injuries and broader PT dysfunction than the patients with the other etiologies. We expect more enrolled patients with minor PT injuries caused by plasmacyte diseases in the future and more studies on the mechanism of how AA transporters were injured by light chains, which would bring us a deeper understanding of the above phenomenon and a potential application of urinary AAs excretion patterns in differential diagnosis of the etiologies of PT injuries.

The diagnosis of renal FS was relatively cumbersome and limited by accurate assessments relied on 24-hour urine collection and renal physiological functional tests,^[12,20,23]^ which proposed a need for an easier and more rapid method to diagnose renal FS. Thus, we established fine diagnostic models of renal FS with all the 21 urinary AAs by machine learning with internal training and testing sets as AUC values of 0.81 in XGBoost algorithm and 0.82 in RF algorithm, respectively. These models were better than the models established by traditional clinical indicators and similar to the models combining both traditional clinical indicators and urinary AAs. Inspiringly, an optimization model based on only four urinary AAs had a similar diagnostic value in renal FS (AUC 0.88), which might provide convenience for clinical application.^[28]^

It was worth noting that we observed an independent correlation between the urinary Pro excretion levels and the decline of eGFR, even after adjusted by age and etiologies. Meanwhile there were significant elevations of urinary BCAAs levels in the patients with renal insufficiency. We also developed a model to predict renal insufficiency based on urinary AAs excretion patterns. These all indicated that aminoaciduria and defects of tubular AA transporters could reflect not only PT injuries but also renal function decline, suggesting they may serve as an indicator or a therapy target for renal insufficiency. AA transporters in proximal tubules not only reabsorb AAs to keep nutrition balance but also regulate local metabolic processes,^[29,30]^ inflammatory reactions,^[31]^ and the para- and trans-cellular transports of other solutes.^[30]^ Previous studies in human and animal models have provided consistent evidence supporting these findings. A randomized trial has proved that supplementary intravenous AAs would reduce the occurrence of AKI induced by cardiology surgery.^[32]^ Impaired reabsorption of BCAAs in proximal tubules may lead to reduction of local BCAAs metabolism and energy generation (Figure 1), which has been shown to be harmful for PT cells in both human and mice, ultimately contributing to kidney injury.^[13,15]^ However, some controversial animal experiments indicated a protection effect in AKI by inhibition of tubular AA transporter SLC6A19 (B^0^AT1) in aristolochic acid-induced nephropathy^[33]^ and diabetic nephropathy,^[34]^ which needs further studies focused on the detailed mechanism.

We noted that specific urinary AAs were associated with the other PT dysfunction like hypouricemia and hypokalemia, indicating potential cross-talk between transport of AAs and the other substrates like urate and potassium in PT cells. Previous studies had showed similar phenomenon. Kugathasan *et al* reported that sodium-glucose cotransporter 2 inhibitors could lead to increasement of urinary AAs excretion in type 1 diabetes patients.^[35]^ These potential crosstalk between different PT transporters might offer new therapeutic targets for not only AAs associated rare diseases such as phenylketonuria,^[36]^ but also for common diseases including renal stones, hyperuricemia and diabetes nephropathy.^[37]^ The potential physiological mechanisms included common regulatory pathways between transport of AAs and the other substrates in proximal tubules such as the membrane-associated proteins and the regulation of urea gradients. For instance, the membrane-associated protein Na^+^/H^+^ exchanger regulatory factor 1 (NHERF1) could regulate the transport of both phosphate and uric acid by interacting with sodium-phosphate cotransporter IIa and urate transporter together.^[38,39]^ Additionally, AA transporters might work as urea antiporters and influence the transport of other substrates by regulation of urea gradients in the proximal straight tubules.^[40]^

It was remarkable that the associations between transporting AAs and the other substrates like urate and potassium showed an obvious gender difference in our study, being more prominent in males than females. Although the precise mechanisms of these gender differences remained unknown, several previous findings might shed some light. Firstly, the distributions of AA transporters in proximal tubules showed gender-related differences, for instance SLC3A1 was more strongly expressed in males than females, while SLC6A19 (B^0^AT1) was more strongly expressed in females versus males.^[41,42]^ Secondly, the functions of AA transporters might be different between males and females. As in cystinuria, a genetic disorder caused by dysfunction of the AA transporter SLC3A1/ SLC7A9 and hyper-excretion of cystine in the urine, both male patients and male mice models were affected more frequently and severely than females with an earlier onset of stone formation and a larger number of stones,^[43,44]^ indicating potential gender-specific functional diversity of SLC3A1/SLC7A9. Additionally, the differential regulatory effects of sex hormones on PT transporters might be involved as the androgen receptor and estrogen receptor α were mainly expressed in the S2 and S3 PT segments, while estrogen receptor β was mainly seen in loop of Henle segments.^[42]^

Our study has several limitations. First, being a retrospective cross-sectional study, we could not avoid the selection bias and were unable to establish a predictive model for renal function evolution and renal prognosis. Additionally, since urinary AA levels detected by LC-MS/MS were not widely used in clinical practice, enrolling more patients and data from external centers in the future would provide more solid evidence for further validation.

In conclusion, this study was the first time mapping the etiology-specific urinary AAs excretion atlases in patients with PT injuries. We also established a fine and simplified machine-learning model for renal FS diagnosis based on only four urinary AAs, and observed significant associations between abnormal urinary excretion of specific AAs with some other PT dysfunction, including hypouricemia and hypokalemia, especially in the male patients.

## Supplementary Information

Supplementary materials are only available at the official site of the journal (www.intern-med.com).

## Supplementary Material

Supplementary Material Details

## References

[1] Kandasamy P, Gyimesi G, Kanai Y, Hediger MA (2018). Amino acid transporters revisited: New views in health and disease. Trends Biochem Sci.

[2] Knol MGE, Wulfmeyer VC, Müller R-U, Rinschen MM (2024). Amino acid metabolism in kidney health and disease. Nat Rev Nephrol.

[3] Makrides V, Camargo SMR, Verrey F (2014). Transport of amino acids in the kidney. Compr Physiol.

[4] Kleta R, Romeo E, Ristic Z, Ohura T, Stuart C, Arcos-Burgos M (2004). Mutations in SLC6A19, encoding B0AT1, cause Hartnup disorder. Nat Genet.

[5] Fairweather SJ, Bröer A, Subramanian N, Tumer E, Cheng Q, Schmoll D (2015). Molecular basis for the interaction of the mammalian amino acid transporters B0AT1 and B0AT3 with their ancillary protein collectrin. J Biol Chem.

[6] Cherqui S, Courtoy PJ (2017). The renal Fanconi syndrome in cystinosis: pathogenic insights and therapeutic perspectives. Nat Rev Nephrol.

[7] Nagamori S, Wiriyasermkul P, Guarch ME, Okuyama H, Nakagomi S, Tadagaki K (2016). Novel cystine transporter in renal proximal tubule identified as a missing partner of cystinuria-related plasma membrane protein rBAT/SLC3A1. Proc Natl Acad Sci U S A.

[8] Kanai Y, Hediger MA (1992). Primary structure and functional characterization of a high-affinity glutamate transporter. Nature.

[9] Boll M, Foltz M, Rubio-Aliaga I, Kottra G, Daniel H (2002). Functional characterization of two novel mammalian electrogenic proton-dependent amino acid cotransporters. J Biol Chem.

[10] Brown JL, Samiy AH, Pitts RF (1961). Localization of amino-nitrogen reabsorption in the nephron of the dog. Am J Physiol Leg Content.

[11] Foreman JW (2019). Fanconi syndrome. Pediatr Clin N Am.

[12] Shi X, Chen Z, Wang J, Wen Y, Zou L, Fei Y (2020). Primary Sjӧgren’s syndrome with renal Fanconi syndrome: Good responses to treatment with glucocorticoids. Semin Arthritis Rheum.

[13] Piret SE, Guo Y, Attallah AA, Horne SJ, Zollman A, Owusu D (2021). Krüppel-like factor 6-mediated loss of BCAA catabolism contributes to kidney injury in mice and humans. Proc Natl Acad Sci U S A.

[14] Piret SE, Mallipattu SK (2023). Transcriptional regulation of proximal tubular metabolism in acute kidney injury. Pediatr Nephrol.

[15] DiMartino S, Revelo MP, Mallipattu SK, Piret SE (2025). Activation of branched chain amino acid catabolism protects against nephrotoxic acute kidney injury. Am J Physiol Renal Physiol.

[16] Oe Y, Vallon V (2023). CRRT 2023 meeting: targeting amino acid transport to improve acute kidney injury outcome. Nephron.

[17] Kurko J, Tringham M, Tanner L, Näntö-Salonen K, Vähä-Mäkilä M, Nygren H (2016). Imbalance of plasma amino acids, metabolites and lipids in patients with lysinuric protein intolerance (LPI). Metabolism.

[18] Riccio E, Pisani A (2014). Fanconi syndrome with lysinuric protein intolerance. Clin Kidney J.

[19] Magen D, Sprecher E, Zelikovic I, Skorecki K (2005). A novel missense mutation in SLC5A2 encoding SGLT2 underlies autosomal-recessive renal glucosuria and aminoaciduria. Kidney Int.

[20] Chen Z, Li J, Shi X, Wang Y, Xia P, Ye W (2021). Clinicopathological characteristics and long-term prognosis of monoclonal immunoglobulin light chain associated Fanconi syndrome. Ther Adv Hematol.

[21] Atanassova SS, Panchev P, Ivanova M (2010). Plasma levels and urinary excretion of amino acids by subjects with renal calculi. Amino Acids.

[22] Maric S, Flüchter P, Guglielmetti LC, Staerkle RF, Sasse T, Restin T (2021). Plasma citrulline correlates with basolateral amino acid transporter LAT4 expression in human small intestine. Clin Nutr.

[23] Vignon M, Javaugue V, Alexander MP, El-Karoui K, Karras A, Roos-Weil D (2017). Current anti-myeloma therapies in renal manifestations of monoclonal light chain-associated Fanconi syndrome: a retrospective series of 49 patients. Leukemia.

[24] Payne RB (1998). Renal tubular reabsorption of phosphate (TmP/GFR): indications and interpretation. Ann Clin Biochem.

[25] Haijes HA, Prinsen HCMT, de Sain-van der Velden MGM, Verhoeven-Duif NM, van Hasselt PM, Jans JJM (2019). Accurate discrimination of Hartnup disorder from other aminoacidurias using a diagnostic ratio. Mol Genet Metab Rep.

[26] Bröer A, Cavanaugh JA, Rasko JEJ, Bröer S (2006). The molecular basis of neutral aminoacidurias. Pflugers Arch.

[27] Javed K, Bröer S (2019). Mice lacking the intestinal and renal neutral amino acid transporter SLC6A19 demonstrate the relationship between dietary protein intake and amino acid malabsorption. Nutrients.

[28] Ye L, Li X, Ghorbani H, Zhou X, Minasyan A, Zheng B (2025). Artificial intelligence in rheumatology: A transformative perspective. J Transl Int Med.

[29] Toback FG, Dodd RC, Maier ER, Havener LJ (1983). Amino acid administration enhances renal protein metabolism after acute tubular necrosis. Nephron.

[30] Pfeffer T, Krug SM, Kracke T, Schürfeld R, Colbatzky F, Kirschner P (2024). Knock-out of dipeptidase CN2 in human proximal tubular cells disrupts dipeptide and amino acid homeostasis and para- and transcellular solute transport. Acta Physiol.

[31] Cao W, Zhang J, Yu S, Gan X, An R (2024). N-acetylcysteine regulates oxalate induced injury of renal tubular epithelial cells through CDKN2B/TGF-β/SMAD axis. Urolithiasis.

[32] Landoni G, Monaco F, Ti LK, Baiardo Redaelli M, Bradic N, Comis M (2024). A randomized trial of intravenous amino acids for kidney protection. N Engl J Med.

[33] Navarro Garrido A, Kim YC, Oe Y, Zhang H, Crespo-Masip M, Good-luck HA (2022). Aristolochic acid-induced nephropathy is attenuated in mice lacking the neutral amino acid transporter B0AT1 (Slc6a19). Am J Physiol Ren Physiol.

[34] Kano Y, Yamaguchi S, Mise K, Kawakita C, Onishi Y, Kurooka N (2024). Inhibition of amino acids influx into proximal tubular cells improves lysosome function in diabetes. Kidney360.

[35] Kugathasan L, Sridhar VS, Lovblom LE, Matta S, Saliba A, Debnath S (2024). Interactive effects of empagliflozin and hyperglycemia on urinary amino acids in individuals with type 1 diabetes. Diabetes.

[36] Wobst HJ, Viader A, Muncipinto G, Hollibaugh R, van Kalken D, Burkhart CT (2024). SLC6A19 inhibition facilitates urinary neutral amino acid excretion and lowers plasma phenylalanine. JCI Insight.

[37] He J, Lin R, Long H, Liang Y, Chen Y (2015). Adsorption characteristics of amino acids on to calcium oxalate. J Colloid Interface Sci.

[38] Cunningham R, Brazie M, Kanumuru S, Xiaofei E, Biswas R, Wang F (2007). Sodium-hydrogen exchanger regulatory factor-1 interacts with mouse urate transporter 1 to regulate renal proximal tubule uric acid transport. J Am Soc Nephrol.

[39] Weinman EJ, Lederer ED (2012). NHERF-1 and the regulation of renal phosphate reabsoption: a tale of three hormones. Am J Physiol Renal Physiol.

[40] Bankir L, Crambert G, Vargas-Poussou R (2024). The SLC6A18 transporter is most likely a Na-dependent Glycine/urea antiporter responsible for urea secretion in the proximal straight tubule: influence of this urea secretion on glomerular filtration rate. Nephron.

[41] Ransick A, Lindström NO, Liu J, Zhu Q, Guo JJ, Alvarado GF (2019). Single-cell profiling reveals sex, lineage, and regional diversity in the mouse kidney. Dev Cell.

[42] Lewis S, Chen L, Raghuram V, Khundmiri SJ, Chou C-L, Yang C-R (2021). “SLC-omics” of the kidney: solute transporters along the nephron. Am J Physiol Cell Physiol.

[43] Eggermann T, Venghaus A, Zerres K (2012). Cystinuria: an inborn cause of urolithiasis. Orphanet J Rare Dis.

[44] Sahota A, Tischfield JA, Goldfarb DS, Ward MD, Hu L (2019). Cystinuria: genetic aspects, mouse models, and a new approach to therapy. Urolithiasis.

