# Supplementary material for: Urinary amino acids patterns enlighten new predictive models for extensive renal proximal tubular injuries and kidney function
Source: J Transl Int Med. 2026 Oct 23;14(5):736–47. doi: 10.1515/jtim-2026-0049 (PMC13726659; doi:10.1515/jtim-2026-0049)
Supplement: Supplementary file 1 [file jtim-2026-0049_sm.pdf]

# Supplementary materials

**Supplementary Table S1: The ratios of abnormal urinary amino acids excretion in the 157 patients with different etiologies**

| Urinary AAs type | All cases ($n$ = 157) | Renal FS ($n$ = 75) | pSS ($n$ = 26) | Drug-induced ($n$ = 17) | Plasmacyte disease ($n$ = 8) | TIO ($n$ = 32) | $P$ |
|---|---|---|---|---|---|---|---|
| Cit | 89(56.7) | 60(80.0) | 18(69.2) | 12(70.6) | 6(75.0) | 18(56.3) | 0.114 |
| Thr | 82(52.2) | 53(70.7) | 12(46.2) | 13(76.5) | 6(75.0) | 18(56.3) | 0.088 |
| Phe | 76(48.4) | 39(52.0) | 10(38.5) | 10(58.8) | 6(75.0) | 11(34.4) | 0.302 |
| Ser | 74(47.1) | 42(56.0) | 10(38.5) | 8(47.1) | 6(75.0) | 12(37.5) | 0.396 |
| Orn | 72(45.9) | 48(64.0) | 11(42.3) | 12(70.6) | 5(62.5) | 9(28.1) | 0.004 |
| Tyr | 71(45.2) | 41(54.7) | 8(30.8) | 12(70.6) | 5(62.5) | 12(37.5) | 0.182 |
| Val | 61(38.9) | 37(49.3) | 8(30.8) | 9(52.9) | 6(75.0) | 8(25.0) | 0.164 |
| Leu | 59(37.6) | 35(46.7) | 8(30.8) | 11(64.7) | 5(62.5) | 6(18.8) | 0.056 |
| Trp | 58(36.9) | 30(40.0) | 6(23.1) | 7(41.2) | 5(62.5) | 15(46.9) | 0.176 |
| Ile | 57(36.3) | 35(46.7) | 10(38.5) | 9(52.9) | 7(87.5) | 4(12.5) | 0.001 |
| Gln | 56(35.7) | 39(52.0) | 9(34.6) | 9(52.9) | 6(75.0) | 6(18.8) | 0.040 |
| Ala | 55(35.0) | 39(52.0) | 9(34.6) | 8(47.1) | 6(75.0) | 7(21.9) | 0.038 |
| Pro | 54(34.4) | 42(56.0) | 7(26.9) | 9(52.9) | 6(75.0) | 9(28.1) | 0.015 |
| Met | 52(33.1) | 28(37.3) | 8(30.8) | 10(58.8) | 4(50.0) | 4(12.5) | 0.007 |
| Asn | 52(33.1) | 40(53.3) | 8(30.8) | 10(58.8) | 6(75.0) | 7(21.9) | 0.059 |
| Gly | 48(30.6) | 33(44.0) | 5(19.2) | 4(23.5) | 3(37.5) | 17(53.1) | 0.098 |
| Lys | 43(27.4) | 34(45.3) | 9(34.6) | 10(58.8) | 4(50.0) | 6(18.8) | 0.034 |
| Glu | 40(25.5) | 28(37.3) | 6(23.1) | 4(23.5) | 4(50.0) | 3(9.4) | 0.143 |
| His | 39(24.8) | 21(28.0) | 4(15.4) | 3(17.6) | 5(62.5) | 9(28.1) | 0.295 |
| Asp | 26(16.6) | 20(26.7) | 3(11.5) | 5(29.4) | 2(25.0) | 3(9.4) | 0.725 |

| | | | | | | | |
|---|---|---|---|---|---|---|---|
| Arg | 21(13.4) | 19(25.3) | 3(11.5) | 5(29.4) | 4(50.0) | 1(3.1) | 0.016 |

Data were presented as *n* (%). AAs: amino acids; FS: Fanconi syndrome; pSS: primary Sjögren's syndrome; TIO: tumor-induced osteomalacia; Ala: alanine; Arg: arginine; Asn: asparagine; Asp: aspartic acid; Cit: citrulline; Gln: glutamine; Glu: glutamic acid; Gly: glycine; His: histidine; Ile: isoleucine; Leu: leucine; Lys: lysine; Met: methionine; Orn: ornithine; Phe: phenylalanine; Pro: proline; Ser: serine; Thr: threonine; Trp: tryptophan; Tyr: tyrosine; Val: valine.

**Supplementary Table S2: The comparisons of the ratios of abnormal urinary AAs excretion between the patients with or without a specific proximal tubular dysfunction.**

| AAs transporter | Urinary AAs type | Hypokalemia | | | Hypophosphatemia | | | Hypouricemia | | | Normoglycemic glycosuria | | |
|---|---|---|---|---|---|---|---|---|---|---|---|---|---|
| | | Yes (*n* = 66) | No (*n* = 87) | *P* | Yes (*n* = 76) | No (*n* = 73) | *P* | Yes (*n* = 42) | No (*n* = 108) | *P* | Yes (*n* = 52) | No (*n* = 100) | *P* |
| SLC36A1/2 | Ala | 31(47.0) | 22(25.3) | 0.005 | 28(36.8) | 21(28.8) | 0.294 | 24(57.1) | 29(26.9) | < 0.001 | 20(38.5) | 34(34.0) | 0.586 |
| | Pro | 28(42.4) | 26(29.9) | 0.108 | 30(39.5) | 20(27.4) | 0.119 | 22(52.4) | 32(29.6) | 0.009 | 21(40.4) | 32(32.0) | 0.303 |
| | Gly | 22(33.3) | 25(28.7) | 0.542 | 32(42.1) | 14(19.2) | 0.002 | 17(40.5) | 30(27.8) | 0.132 | 18(34.6) | 30(30.0) | 0.561 |
| SLC6A19/20 | Leu | 30(45.5) | 28(32.2) | 0.094 | 26(34.2) | 29(39.7) | 0.486 | 25(59.5) | 33(30.6) | 0.001 | 20(38.5) | 38(38.0) | 0.956 |
| | Trp | 22(33.3) | 35(40.2) | 0.382 | 31(40.8) | 23(31.5) | 0.306 | 20(47.6) | 36(33.3) | 0.104 | 21(40.4) | 36(36.0) | 0.596 |
| | His | 17(25.8) | 20(23.0) | 0.692 | 22(28.9) | 14(19.2) | 0.164 | 11(26.2) | 25(23.1) | 0.695 | 13(25.0) | 25(25.0) | 1.000 |
| | Met | 23(34.8) | 28(32.2) | 0.729 | 21(27.6) | 25(34.2) | 0.382 | 19(45.2) | 31(28.7) | 0.054 | 17(32.7) | 34(34.0) | 0.871 |
| | Thr | 39(59.1) | 41(47.1) | 0.142 | 46(60.5) | 32(43.8) | 0.041 | 28(66.7) | 53(49.1) | 0.052 | 29(55.8) | 51(51.0) | 0.576 |
| | Val | 28(42.4) | 32(36.8) | 0.479 | 28(36.8) | 27(37.0) | 0.985 | 23(54.8) | 36(33.3) | 0.016 | 23(44.2) | 38(38.0) | 0.457 |
| | Tyr | 32(48.5) | 37(42.5) | 0.463 | 36(47.4) | 29(39.7) | 0.347 | 27(64.3) | 41(38.0) | 0.004 | 26(50.0) | 43(43.0) | 0.411 |
| | Ile | 30(45.5) | 26(29.9) | 0.048 | 27(35.5) | 27(37.0) | 0.853 | 25(59.5) | 31(28.7) | < 0.001 | 21(40.4) | 35(35.0) | 0.514 |
| | Phe | 33(50.0) | 41(47.1) | 0.725 | 35(46.1) | 35(47.9) | 0.817 | 26(61.9) | 47(43.5) | 0.043 | 26(50.0) | 48(48.0) | 0.815 |
| | Ser | 34(51.5) | 38(43.7) | 0.336 | 38(50.0) | 32(43.8) | 0.451 | 25(59.5) | 46(42.6) | 0.062 | 29(55.8) | 43(43.0) | 0.135 |
| | Asn | 30(45.5) | 21(24.1) | 0.006 | 30(39.5) | 19(26.0) | 0.081 | 27(64.3) | 24(22.2) | < 0.001 | 19(36.5) | 32(32.0) | 0.574 |
| | Gln | 32(48.5) | 23(26.4) | 0.005 | 31(40.8) | 21(28.8) | 0.124 | 25(59.5) | 30(27.8) | < 0.001 | 21(40.4) | 34(34.0) | 0.437 |
| | Cit | 46(69.7) | 42(48.3) | 0.008 | 50(65.8) | 35(47.9) | 0.028 | 33(78.6) | 54(50.0) | 0.001 | 29(55.8) | 58(58.0) | 0.792 |

| | | | | | | | | | | | | |
|---|---|---|---|---|---|---|---|---|---|---|---|---|
| SLC3A1 /SLC7A9 | Lys | 26(39.4) | 17(19.5) | 0.007 | 25(32.9) | 16(21.9) | 0.134 | 24(57.1) | 19(17.6) | < 0.001 | 16(30.8) | 27(27.0) | 0.625 |
| | Arg | 10(15.2) | 11(12.6) | 0.655 | 13(17.1) | 7(9.6) | 0.178 | 12(28.6) | 9(8.3) | 0.001 | 8(15.4) | 13(13.0) | 0.686 |
| | Orn | 35(53.0) | 36(41.4) | 0.152 | 37(48.7) | 31(42.5) | 0.446 | 28(66.7) | 42(38.9) | 0.002 | 27(51.9) | 43(43.0) | 0.295 |
| SLC1A1 | Asp | 17(25.8) | 8(9.2) | 0.006 | 14(18.4) | 10(13.7) | 0.433 | 11(26.2) | 14(13.0) | 0.051 | 11(21.2) | 15(15.0) | 0.339 |
| | Glu | 27(40.9) | 13(14.9) | < 0.001 | 22(28.9) | 17(23.3) | 0.432 | 18(42.9) | 22(20.4) | 0.005 | 15(28.8) | 25(25.0) | 0.609 |

Data were presented as *n* (%). AAs: amino acids; Ala: alanine; Arg: arginine; Asn: asparagine; Asp: aspartic acid; Cit: citrulline; Gln: glutamine; Glu: glutamic acid; Gly: glycine; His: histidine; Ile: isoleucine; Leu: leucine; Lys: lysine; Met: methionine; Orn: ornithine; Phe: phenylalanine; Pro: proline; Ser: serine; Thr: threonine; Trp: tryptophan; Tyr: tyrosine; Val: valine.

**Supplementary Table S3a: Regression analysis of urinary amino acids excretion levels with serum biochemical indicators in the female patients.**

| AA transporter | Urinary AAs type | Serum potassium | | Serum phosphate | | Serum uric acid | |
|---|---|---|---|---|---|---|---|
| | | Correlation coefficient | *P* | Correlation coefficient | *P* | Correlation coefficient | *P* |
| SLC36A1/2 | Ala | -0.043 | 0.695 | -0.108 | 0.321 | -0.220 | 0.043 |
| | Pro | 0.069 | 0.531 | -0.075 | 0.488 | -0.053 | 0.631 |
| | Gly | 0.106 | 0.333 | -0.356 | 0.001 | -0.058 | 0.595 |
| SLC6A19/20 | Leu | 0.020 | 0.854 | -0.062 | 0.570 | -0.202 | 0.064 |
| | Trp | 0.122 | 0.267 | -0.152 | 0.159 | -0.094 | 0.390 |
| | His | 0.064 | 0.563 | -0.044 | 0.686 | -0.014 | 0.895 |
| | Met | 0.081 | 0.462 | -0.014 | 0.897 | -0.120 | 0.273 |
| | Thr | -0.027 | 0.805 | -0.130 | 0.229 | -0.199 | 0.068 |
| | Val | 0.057 | 0.604 | -0.039 | 0.720 | -0.159 | 0.146 |
| | Tyr | 0.069 | 0.530 | -0.179 | 0.098 | -0.174 | 0.111 |
| | Ile | -0.023 | 0.836 | 0.106 | 0.327 | -0.139 | 0.204 |
| | Phe | 0.111 | 0.313 | -0.043 | 0.693 | -0.161 | 0.140 |
| | Ser | 0.008 | 0.942 | -0.114 | 0.295 | -0.169 | 0.122 |
| | Asn | -0.047 | 0.667 | -0.109 | 0.317 | -0.287 | 0.008 |
| | Gln | -0.053 | 0.630 | -0.128 | 0.236 | -0.268 | 0.013 |
| | Cit | -0.138 | 0.207 | -0.157 | 0.147 | -0.279 | 0.010 |
| SLC3A1 | Lys | -0.100 | 0.362 | -0.143 | 0.187 | -0.265 | 0.014 |
| /SLC7A9 | Arg | 0.030 | 0.789 | -0.073 | 0.502 | -0.076 | 0.488 |
| | Orn | -0.044 | 0.691 | -0.150 | 0.166 | -0.174 | 0.111 |
| SLC1A1 | Asp | -0.177 | 0.105 | -0.037 | 0.731 | -0.195 | 0.074 |
| | Glu | -0.113 | 0.305 | -0.067 | 0.537 | -0.161 | 0.141 |

AAs: amino acids; Ala: alanine; Arg: arginine; Asn: asparagine; Asp: aspartic acid; Cit: citrulline; Gln: glutamine; Glu: glutamic acid; Gly: glycine; His: histidine; Ile: isoleucine; Leu: leucine; Lys: lysine; Met: methionine; Orn: ornithine; Phe: phenylalanine; Pro: proline; Ser: serine; Thr: threonine; Trp: tryptophan; Tyr: tyrosine; Val: valine.

**Supplementary Table S3b: Regression analysis of urinary amino acids excretion levels with serum biochemical indicators in the male patients.**

| AA transporter | Urinary AAs type | Serum potassium | | Serum phosphate | | Serum uric acid | |
|---|---|---|---|---|---|---|---|
| | | Correlation coefficient | *P* | Correlation coefficient | *P* | Correlation coefficient | *P* |
| SLC36A1/2 | Ala | -0.288 | 0.037 | -0.248 | 0.066 | -0.449 | 0.001 |
| | Pro | -0.306 | 0.026 | -0.179 | 0.186 | -0.410 | 0.003 |
| | Gly | -0.228 | 0.101 | -0.272 | 0.043 | -0.203 | 0.154 |
| SLC6A19/20 | Leu | -0.361 | 0.008 | 0.032 | 0.814 | -0.457 | 0.001 |
| | Trp | -0.334 | 0.015 | -0.126 | 0.355 | -0.361 | 0.009 |
| | His | -0.209 | 0.133 | -0.463 | < 0.001 | -0.288 | 0.041 |
| | Met | -0.193 | 0.166 | 0.011 | 0.937 | -0.361 | 0.009 |
| | Thr | -0.477 | < 0.001 | -0.165 | 0.225 | -0.426 | 0.002 |
| | Val | -0.295 | 0.032 | -0.043 | 0.751 | -0.369 | 0.008 |
| | Tyr | -0.356 | 0.009 | -0.058 | 0.669 | -0.430 | 0.002 |
| | Ile | -0.241 | 0.082 | 0.087 | 0.523 | -0.326 | 0.020 |
| | Phe | -0.343 | 0.012 | 0.009 | 0.946 | -0.380 | 0.006 |
| | Ser | -0.439 | 0.001 | -0.109 | 0.422 | -0.327 | 0.019 |
| | Asn | -0.358 | 0.009 | -0.226 | 0.094 | -0.511 | 0.000 |
| | Gln | -0.340 | 0.013 | -0.198 | 0.144 | -0.419 | 0.002 |
| | Cit | -0.461 | 0.001 | -0.193 | 0.155 | -0.460 | 0.001 |
| SLC3A1 | Lys | -0.372 | 0.006 | -0.145 | 0.287 | -0.402 | 0.003 |
| /SLC7A9 | Arg | -0.200 | 0.151 | -0.158 | 0.246 | -0.397 | 0.004 |
| | Orn | -0.153 | 0.273 | -0.171 | 0.208 | -0.355 | 0.011 |
| SLC1A1 | Asp | -0.309 | 0.024 | 0.061 | 0.656 | -0.383 | 0.006 |
| | Glu | -0.237 | 0.087 | -0.081 | 0.555 | -0.328 | 0.019 |

AAs: amino acids; Ala: alanine; Arg: arginine; Asn: asparagine; Asp: aspartic acid; Cit: citrulline; Gln: glutamine; Glu: glutamic acid; Gly: glycine; His: histidine; Ile: isoleucine; Leu: leucine; Lys: lysine; Met: methionine; Orn: ornithine; Phe: phenylalanine; Pro: proline; Ser: serine; Thr: threonine; Trp: tryptophan; Tyr: tyrosine; Val: valine.

**Supplementary Table S4: Regression analysis of urinary amino acids excretion levels with serum biochemical indicators.**

| AAs transporter | Urinary AAs type | Serum potassium | | Serum phosphate | | Serum uric acid | |
|---|---|---|---|---|---|---|---|
| | | Correlation coefficient | *P* | Correlation coefficient | *P* | Correlation coefficient | *P* |
| SLC36A1/2 | Ala | -0.162 | 0.043 | -0.024 | 0.764 | 0.035 | 0.219 |
| | Pro | -0.193 | 0.015 | -0.017 | 0.836 | -0.140 | 0.281 |
| | Gly | -0.16 | 0.045 | 0.032 | 0.694 | -0.016 | 0.531 |
| SLC6A19/20 | Leu | -0.091 | 0.256 | -0.027 | 0.740 | 0.028 | 0.188 |
| | Trp | -0.066 | 0.409 | -0.089 | 0.268 | 0.062 | 0.469 |
| | His | -0.066 | 0.412 | -0.093 | 0.248 | 0.129 | 0.281 |
| | Met | -0.111 | 0.167 | -0.063 | 0.435 | -0.057 | 0.125 |
| | Thr | -0.102 | 0.205 | 0.007 | 0.933 | 0.039 | 0.563 |
| | Val | -0.135 | 0.092 | -0.047 | 0.561 | -0.068 | 0.250 |
| | Tyr | -0.040 | 0.618 | -0.072 | 0.367 | 0.047 | 0.375 |
| | Ile | -0.102 | 0.202 | 0.007 | 0.935 | -0.074 | 0.125 |
| | Phe | -0.069 | 0.389 | -0.121 | 0.130 | 0.016 | 0.344 |
| | Ser | -0.029 | 0.721 | 0.011 | 0.892 | 0.070 | 0.375 |
| | Asn | -0.125 | 0.119 | -0.010 | 0.902 | 0.049 | 0.219 |
| | Gln | -0.106 | 0.187 | -0.076 | 0.345 | 0.007 | 0.188 |
| | Cit | -0.067 | 0.407 | -0.005 | 0.950 | 0.053 | 0.563 |
| SLC3A1 | Lys | -0.074 | 0.358 | -0.033 | 0.684 | 0.104 | 0.188 |
| /SLC7A9 | Arg | -0.159 | 0.047 | -0.055 | 0.496 | -0.035 | 0.031 |
| | Orn | 0.024 | 0.767 | 0.003 | 0.974 | 0.041 | 0.281 |
| SLC1A1 | Asp | -0.019 | 0.816 | -0.061 | 0.449 | -0.125 | 0.094 |
| | Glu | -0.093 | 0.245 | -0.050 | 0.530 | 0.005 | 0.094 |

AAs: amino acids; Ala: alanine; Arg: arginine; Asn: asparagine; Asp: aspartic acid; Cit: citrulline; Gln: glutamine; Glu: glutamic acid; Gly: glycine; His: histidine; Ile: isoleucine; Leu: leucine; Lys: lysine; Met: methionine; Orn: ornithine; Phe: phenylalanine; Pro: proline; Ser: serine; Thr: threonine; Trp: tryptophan; Tyr: tyrosine; Val: valine.

(A) (B) (C) (D) (E) (F) (G) (H)

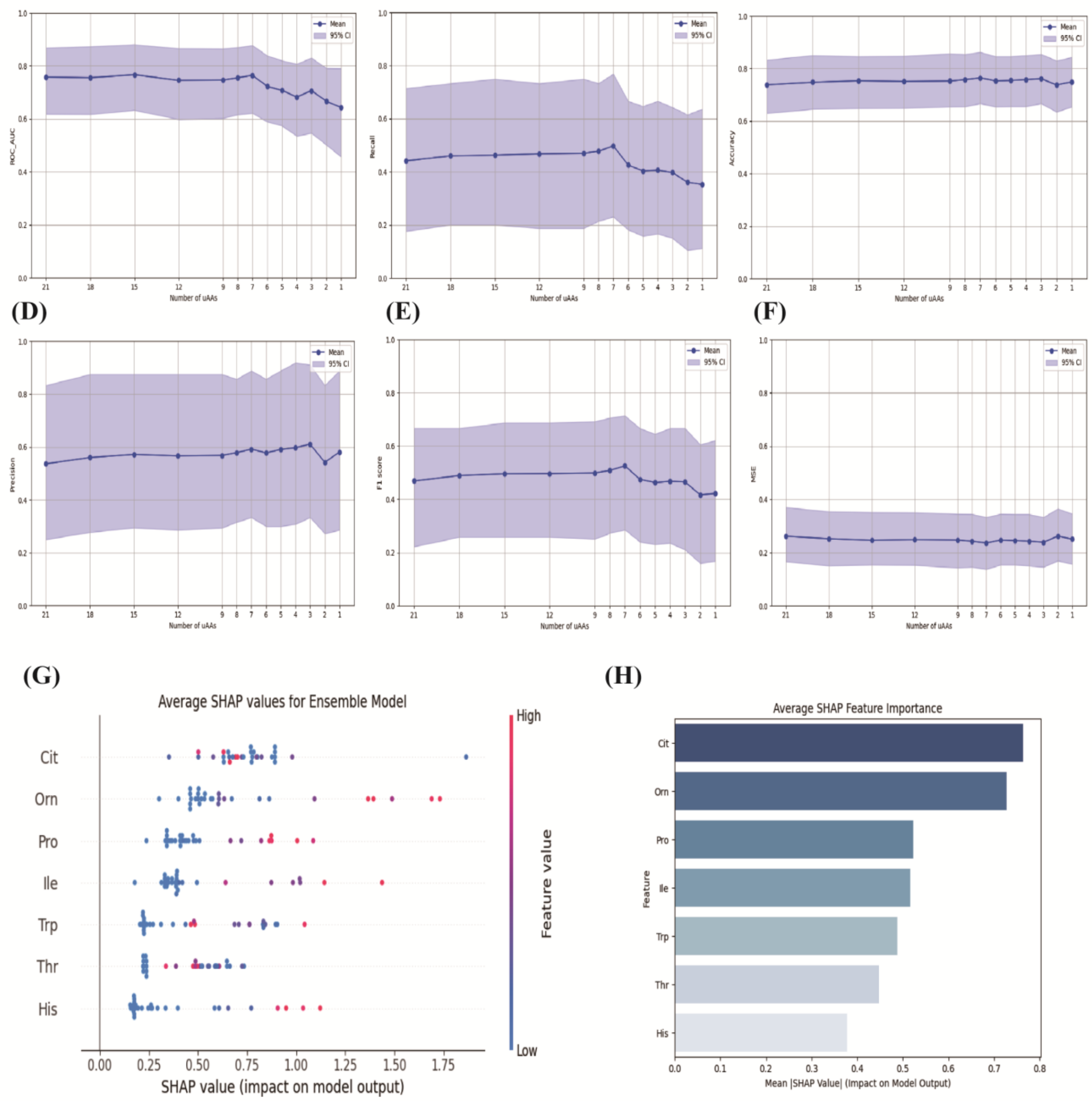


(I)

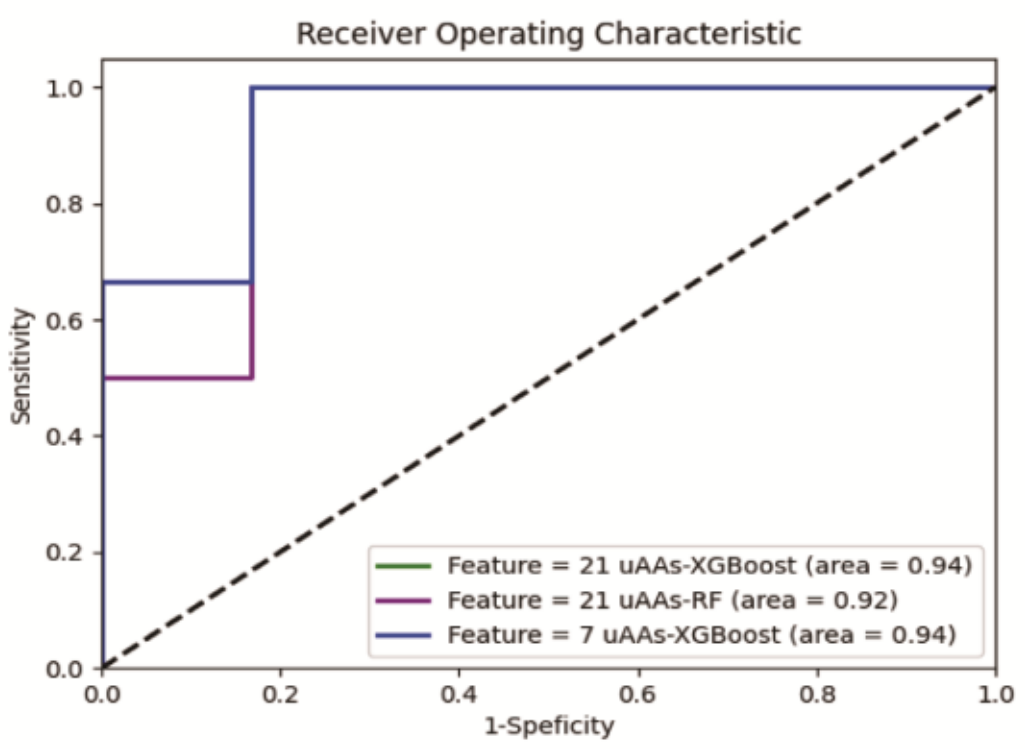


(J)

| Model | AUC | Precision | Accuracy | F1 score | Recall | MSE |
|---|---|---|---|---|---|---|
| 21 uAAs-XGBoost | 0.94 | 1.00 | 0.93 | 0.80 | 0.67 | 0.07 |
| 21 uAAs-RF | 0.94 | 1.00 | 0.93 | 0.80 | 0.67 | 0.07 |
| 7 uAAs-XGBoost | 0.92 | 0.60 | 0.75 | 0.75 | 1.00 | 0.13 |

**Supplementary Figure S1: The predictive model of eGFR < 60 mL/min/1.73 m$^2$ with uAAs through machine learning.** The models were constructed using the XGBoost or RF machine learning algorithm with uAAs. (A-F) The changes in the model performance metrics including AUC (A), Recall (B), Accuracy (C), Precision (D), F1 Score (E), and MSE (F) during optimizing model features with different combinations of uAAs using XGBoost machine learning algorithm. The optimizing process showed that only seven uAAs (Cit, Orn, Pro, Ile, Trp, Thr, and His) could reach a similar diagnostic value as using all the 21 uAAs. (G-H) The SHAP summary plot of the XGBoost model with the above seven uAAs. Each dot represents an individual patient from the dataset. Red represents the higher SHAP value of specific features; blue represents the lower SHAP value of specific features. The higher the SHAP values, the greater the risk of eGFR decline. (I-J) The model performance metrics of different models in predicting eGFR < 60 mL/min/1.73 m$^2$ with machine learning. eGFR: estimated glomerular filtration rate; uAAs: urinary amino acids; XGBoost: eXtreme Gradient Boosting; RF: random forest; AUC: area under the curve; MSE: mean squared error; SHAP: SHapley Additive exPlanation; Cit: citrulline; Orn: ornithine; Pro: proline; Ile: isoleucine; Trp: tryptophan; Thr: threonine; His: histidine.
